# Solitary Fibrous Tumors of the Submandibular Gland: A Case Report

**DOI:** 10.1002/ccr3.71665

**Published:** 2025-12-15

**Authors:** Maryam Garousi, Hossein Gandomkar, Masoud Maleki, Fateme Erteghaee, Farzad Yazdani, Raana Ahmadian

**Affiliations:** ^1^ Department of Radiation Oncology, School of Medicine Iran University of Medical Sciences Tehran Iran; ^2^ Department of General Surgery, School of Medicine Tehran University of Medical Sciences Tehran Iran; ^3^ Department of General Surgery, School of Medicine Iran University of Medical Sciences Tehran Iran; ^4^ Department of Pathology, School of Medicine Tehran University of Medical Sciences Tehran Iran

**Keywords:** case report, soft tissue tumors, solitary fibrous tumors, submandibular mass

## Abstract

Solitary fibrous tumors (SFTs) are uncommon neoplasms that arise from the pleura. Although they can appear in various extrathoracic areas, they are uncommon in the submandibular gland. This paper reports a 37‐year‐old woman with a spherical, hypoechogenic mass measuring 13 × 37 mm and having a regular boundary on the left side of the submandibular gland. The lesion was surgically excised with a less than 1 mm margin. Following histopathological and immunohistochemical examination, the diagnosis of SFT was confirmed. Staining showed that the lesion was negative for vimentin, AE3/CKAE1, calponin, SOX10, desmin, and S‐100 and diffusely positive for CD34, CD99 (MIC‐2), SMA, BCL2, and STAT‐6. Patient underwent postoperative radiation and was observed for one year without showing any symptoms of metastases or recurrence. The study indicates that SFTs of the submandibular gland should be considered in the differential diagnosis of submandibular soft‐tissue tumors, despite their rarity.

## Key Clinical Message

SFT is an uncommon tumor that can be either benign or malignant. Immunohistochemical studies are beneficial in diagnosing this tumor. The suggested approach is surgical excision with negative margins. This case will help treat an uncommon condition.

## 1 Introduction

Soft tissue tumors comprise a group of neoplasms leading to various challenges in diagnosis and treatment. As they are rare, physicians often have limited experience in managing these tumors [1]. Rare mesenchymal tumors known as solitary fibrous tumors (SFTs) can develop in the body's soft tissues [2].

The pleura is the most frequent location for SFTs, accounting for more than 30% of cases; however, they have been discovered in many regions of the body. The meninges (27%), the abdominal cavity (20%), the trunk (10%), the extremities (8%), and the head and neck area (5%) are additional common sites [3]. Although SFT in the salivary glands is highly unusual, manifesting in the submandibular area is even less frequent [4, 5]. Pathological examination—including immunohistochemical and molecular analysis—is crucial for diagnosing SFTs. For SFT analysis, the presence of CD34 is an essential marker, and most patients show diffuse positivity for BCL‐2, STAT6, and CD99 [6]. The most suitable course of treatment is removal by surgery with negative margins, which typically contributes to a good prognosis [7]. In cases of highly vascular tumors, preoperative embolization can be used [8]. For tumors with malignant histological characteristics or those that have not been entirely removed, postoperative radiation and/or chemotherapy may be considered [9].

Now we introduce a case of SFT from the left submandibular gland and discuss its clinical, histopathological, immunohistochemical, and therapeutic aspects.

## 2 Case History and Examination

A 37‐year‐old female presented with an asymptomatic mass that had been gradually increasing in size over 6 months. Physical examination indicated a localized, firm, non‐tender mass in the left submandibular area. Other physical examinations were normal, and she did not mention any family history of any specific disease.

## 3 Differential Diagnosis, Investigations, and Treatment

Ultrasonography (US) showed a 13 × 37 mm, round, hypoechogenic mass with a regular border in the left submandibular region. No additional imaging studies were performed. Due to financial constraints, it was not possible to perform additional imaging on the patient. An incisional biopsy was performed, which showed a spindle cell neoplasm suggestive of a benign tumor.

The lesion was surgically excised with a margin of less than 1 mm. The excised specimen included the tumor and three ipsilateral neck lymph nodes.

In the pathology report, macroscopic examination shows an ovaloid, well‐circumscribed, cream‐colored, rubbery mass, measuring 35 × 30 × 20 mm.

**FIGURE 1:**
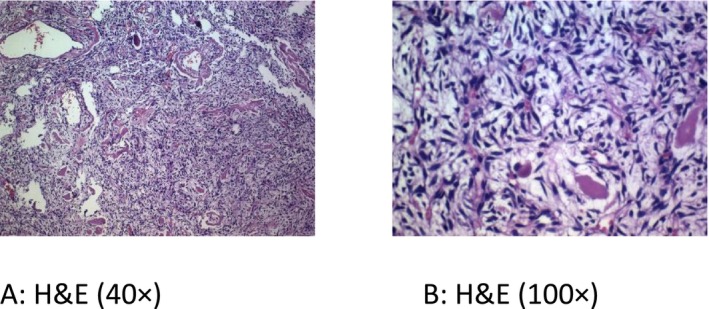
Microscopic analysis reveals ovaloid to spindle‐shaped tumoral cells that are rather monomorphic, have eosinophilic cytoplasm, and undefined boundaries that are randomly or in short fascicles encircling a rich vascular network with perivascular hyalinization and a staghorn structure (The sample image at ×40 magnification after hematoxylin and eosin staining is shown in Figure A, and at ×100 magnification in Figure B).

Microscopic examination shows rather monomorphic ovaloid to spindle tumoral cells with eosinophilic cytoplasm (Figure 1).

**FIGURE 2:**
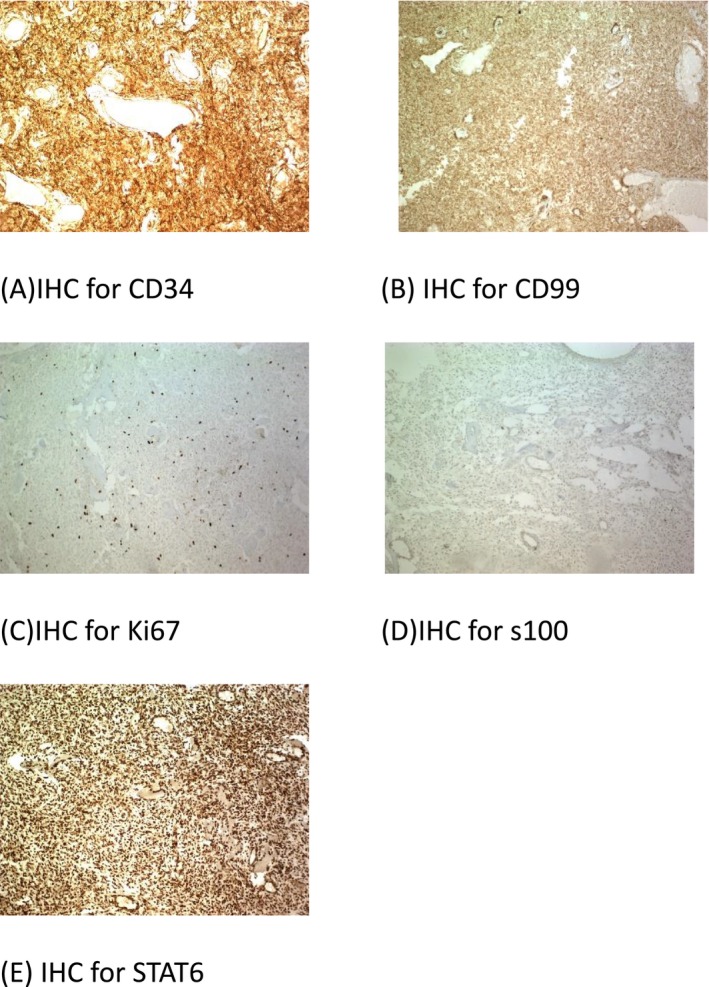
The tumoral cells show immunoreactivity for CD34 (A), CD99 (B), S100 (D), and STAT6 (E) and a low proliferation index (C).

Immunohistochemical (IHC) studies showed that tumor cells stained diffusely for CD34, CD99, BCL2, vimentin, and STAT‐6, and patchily for SMA and Ki‐67 with an index of 10%. IHC was negative for AE3/CKAE1, calponin, SOX10, desmin, and S‐100 (Figure 2). Based on these findings, especially the diffuse staining for STAT‐6 and CD34, and the negative staining for desmin to rule out muscular tumor, as well as negative results for S100 to exclude schwannoma and neurofibroma, the diagnosis of solitary fibrous tumor was confirmed. The three lymph nodes showed reactive changes. Considering the close margin, which was reported to be less than 1 mm in the pathology review and according to the initial location of the tumor, recurrence and re‐surgery would be cosmetically problematic, this patient was referred to the multidisciplinary tumor board, and radiotherapy was recommended in order to close the margin. After 6 weeks of surgery, 60 Gy/30 fractions of adjuvant radiation (3 DC) was administered.

## 4 Outcome and Follow‐Up

No metastasis or relapse occurred throughout the patient's 12‐month follow‐up.

## 5 Discussion

Only 2% of all soft tissue masses are solitary fibrous tumors (SFTs). Extra‐thoracic incidence, including head and neck regions, is quite uncommon, comprising approximately 10% of cases [1, 10].

Klemperer and Rabin first described solitary fibrous tumors (SFTs) as tumors of the pleura in 1931 [11]. However, numerous anatomical locations, such as the paranasal sinuses, larynx, thyroid, pelvis, sublingual gland, cervix, ovary, kidney, tongue, and skin, have been reported to exhibit SFTs [12].

Usually, SFTs are benign but malignant features like metastatic disease are present in 10%–15% of extrapleural SFTs [13].

Histological characteristics includinghigh cellularity, pleomorphism, infiltrative boundaries, necrosis, and more than four mitoses per ten high‐power fields are linked to malignant behavior. Aggressive tumors tend to be larger and located in regions such as the pleura, mediastinum, abdomen, pelvis, or retroperitoneum. Metastasis has been documented in the liver, lungs, and bones, while being uncommon in SFTs [14].

Notably, numerous histological characteristics of SFTs lead to a broad differential diagnosis and frequent misunderstandings. Differential diagnoses include cellular pleomorphic adenoma, myoepithelioma, schwannoma, neurofibroma, benign fibrous histiocytoma, nodular fasciitis, fibromatosis, myofibroblastoma, meningioma, fibrosarcoma, spindle cell squamous cell carcinoma, spindle cell melanoma, Kaposi sarcoma, and monophasic synovial sarcoma [15].

Imaging techniques like MRIs, CT scans, and ultrasounds cannot confirm a diagnosis, as SFTs are tumors with unusual clinical symptoms. Benign SFTs typically emerge as spherical, well‐defined masses in soft tissue areas not constrained by bones on MRI or CT scans. Therefore, it is imperative to differentiate SFT from other body tumors such as pleomorphic adenoma, mucocele, fibrous hyperplasia, lipoma, neurofibroma, neurilemmoma, and leiomyoma [16].

Immunohistochemical findings, especially CD34 positivity, support the mesenchymal origin of SFTs [15]. With a sensitivity of 98% and a specificity of 85%, STAT6 (signal transducer and activator of transcription 6) is an extremely sensitive and specific marker for SFT, while CD34, CD99, and BCL2 are also frequently expressed [17, 18]. Positive rates for CD34 and CD99 reach 90%–95% and 70%–75%, respectively. Generally, SFTs are non‐reactive for CD117, factor VIII, actin, or epithelial membrane antigen. Ki‐67 expression is typically modest, with nuclear positivity occurring in 2%–3% of cells [19].

Demicco et al. evaluated STAT6 expression in 1781 non‐SFT mesenchymal tumors and discovered that nuclear expression was present in just 4% of the cases. SFTs primarily show nuclear STAT6 positivity, unlike other tumors that display both nuclear and cytoplasmic staining [2].

Other differential diagnoses may be ruled out if pan‐cytokeratin and myoepithelial markers like calponin and S100 protein are absent [20].

Because SFTs contain hemangiopericytoma‐like patches, it can be challenging to distinguish SFT from hemangiopericytoma. In contrast to solitary fibrous tumors (SFT), which exhibit spindle‐shaped cellular features, this tumor is made up of cells with round or oval nuclei and blood vessels with thin walls that are formed by cells that are still not completely formed. In addition, the vascular pattern in hemangiopericytoma may be more diffuse than in SFT [21].

Complete surgical resection with negative margins is the backbone of treatment, which can be associated with a good prognosis. In cases of inadequate resection or malignant histological findings, postoperative radiation and/or chemotherapy may be administered.

As recurrence has been reported even after 113 months, long‐term monitoring is highly advised [20].

In our case due to close surgical margins and tumor location, adjuvant radiotherapy was recommended after multidisciplinary tumor board consultation.

## 6 Conclusion

Solitary fibrous tumors (SFTs) should be considered in the differential diagnosis of soft tissue tumors in the head and neck area. It can be challenging to differentiate SFT from other spindle cell neoplasms. Histopathological characterization and immunohistochemical staining, particularly CD34 in combination with Bcl‐2 and STAT6, are the basis for diagnosing this tumor. Careful examination is necessary for areas with focused atypia. Even though surgical excision is still the backbone of treatment, long‐term monitoring is essential because of the possibility of malignant transformation and recurrence.

## Author Contributions

**Raana Ahmadian:** conceptualization, data curation, funding acquisition, project administration, writing – original draft, writing – review and editing. **Maryam Garousi:** data curation, formal analysis, writing – original draft. **Masoud Maleki:** data curation, formal analysis, writing – review and editing. **Fateme Erteghaee:** conceptualization, project administration, writing – review and editing. **Farzad Yazdani:** conceptualization, writing – review and editing. **Hossein Gandomkar:** conceptualization, data curation, formal analysis.

## Funding

The authors have nothing to report.

## Ethics Statement

The patient provided us with a written statement of informed consent for the use of photographs and publication of case facts. The instance described in this publication does not report a new study that needed IRB approval, nor does it contain patient‐identifying information.

## Conflicts of Interest

The authors declare no conflicts of interest.

## Supporting information


**Appendix S1:** ccr371665‐sup‐0001‐AppendixS1.docx.

## Data Availability

The data that support the findings of this case report are not publicly available due to privacy and ethical restrictions. However, they are available from the corresponding author upon reasonable request.
