# Supplementary material for: Solitary Fibrous Tumors of the Submandibular Gland: A Case Report
Source: Clin Case Rep. 2025 Dec 15;13(12):e71665. doi: 10.1002/ccr3.71665 (PMC12705490; doi:10.1002/ccr3.71665)
Supplement: Supplementary file 1 — Appendix S1: ccr371665‐sup‐0001‐AppendixS1.docx. [file CCR3-13-e71665-s001.docx]

**Author Contributions:**

**Maryam Garousi**: data curation, formal analysis, writing – original draft.

**Masoud Maleki:** data curation, formal analysis, writing – review and editing.

**Fateme Erteghaee :** conceptualization, project administration, writing – review and editing

**Farzad Yazdan :** conceptualization , writing – review and editing

**Hossein Gandomkar:** review and editing

**Raana ahmadian:** conceptualization, data curation, funding acquisition, project administration, writing – original draft, writing – review and editing.
